# Malignant Hypercalcemia Revealing Pulmonary Sarcoidosis

**DOI:** 10.7759/cureus.64454

**Published:** 2024-07-13

**Authors:** Mohamed Lakhal, Salaheddine Kebbara, Afaf Thouil, Hatim Kouismi

**Affiliations:** 1 Department of Respiratory Diseases, Research, and Medical Sciences Laboratory, Mohammed VI University Hospital, Oujda, MAR

**Keywords:** angiotensine converting enzyme, corticosteroids, pulmonary sarcoidosis, histology, hypercalcemia

## Abstract

Sarcoidosis is a systemic disorder characterized by the development of granulomatous inflammation in various organs of the body. Hypercalcemia is one of its manifestations. We present a case of malignant hypercalcemia in a 77-year-old man, diagnosed as sarcoidosis following multiple assessments and histological confirmation showing noncaseating granulomas. In the absence of an etiological diagnosis, treatment was initially based on hydration, diuretics, and bisphosphonate. Glucocorticoid therapy can be the standard treatment for hypercalcemia related to sarcoidosis.

## Introduction

Sarcoidosis is a systemic granulomatosis of unknown etiology. It can be difficult to investigate due to its relative rarity, the lack of sensitive and precise diagnostic testing, and variable appearance that may indicate several etiologies [1-2].

Clinical symptoms, computer imaging, physiological investigations, pathological examinations (indicating noncaseating granuloma), and the lack of mycobacterial or fungal infections are the main factors used in the diagnosis of non-specific sarcoidosis [3].

Often known for its generally extensive lung involvement [4], sarcoidosis rarely induces severe hypercalcemia as a challenging condition [5].

We present a case of pulmonary sarcoidosis revealed by malignant hypercalcemia in a 77-year-old man, which was diagnosed thanks to a thorough workup including radiological evaluation and multiple bronchial biopsies. We highlight the diagnostic dilemma of sarcoidosis, namely, its varying clinical presentation that can mimic various illnesses.

## Case presentation

This case is about a 77-year-old man who presented himself to the emergency department with a disturbed consciousness. While he had no family history, he did have a personal history of chronic smoking and ischemic heart disease. Examination revealed a normal blood pressure, a BMI of 18.16 kg/m2, and tachycardia at 110 beats/min (bpm) with regular heart sounds, while pulmonary auscultation revealed bibasilar crackles. Hypercalcemia was the main laboratory abnormality, with a level of 3.15 mmol/L (Table 1). The phosphorous level was normal (1.36 mmol/L) (Table 1). The renal function test showed moderate renal failure (baseline creatinine = 20 mg/L, glomerular filtration rate (GFR) = 35 ml/min/1.73m2) (Table 1). He was started on intravenous hydration (3 L/m2) for 24 hours with pamidronate (30 mg/4 hours) and forced diuresis by loop diuretics, which allowed a normalization of consciousness and calcemia in two days.

**Table 1:** Our patient's biological values compared with normal laboratory values

	Our patient	Normal laboratory values
Calcemia	3.15 mmol/l	2.20-2.60 mmol/L
Phosphoremia	1.36 mmol/l	0.8-1.45 mmol/L
Serum creatinine	176 umol/l	88-150 µmol/L
Creatinine clearance	35 ml/min/1.73m2	90-140 mL/min/1.73m^2^
Angiotensin-converting enzyme	122 IU/l	20 – 70 UI/L

**Figure 1:**
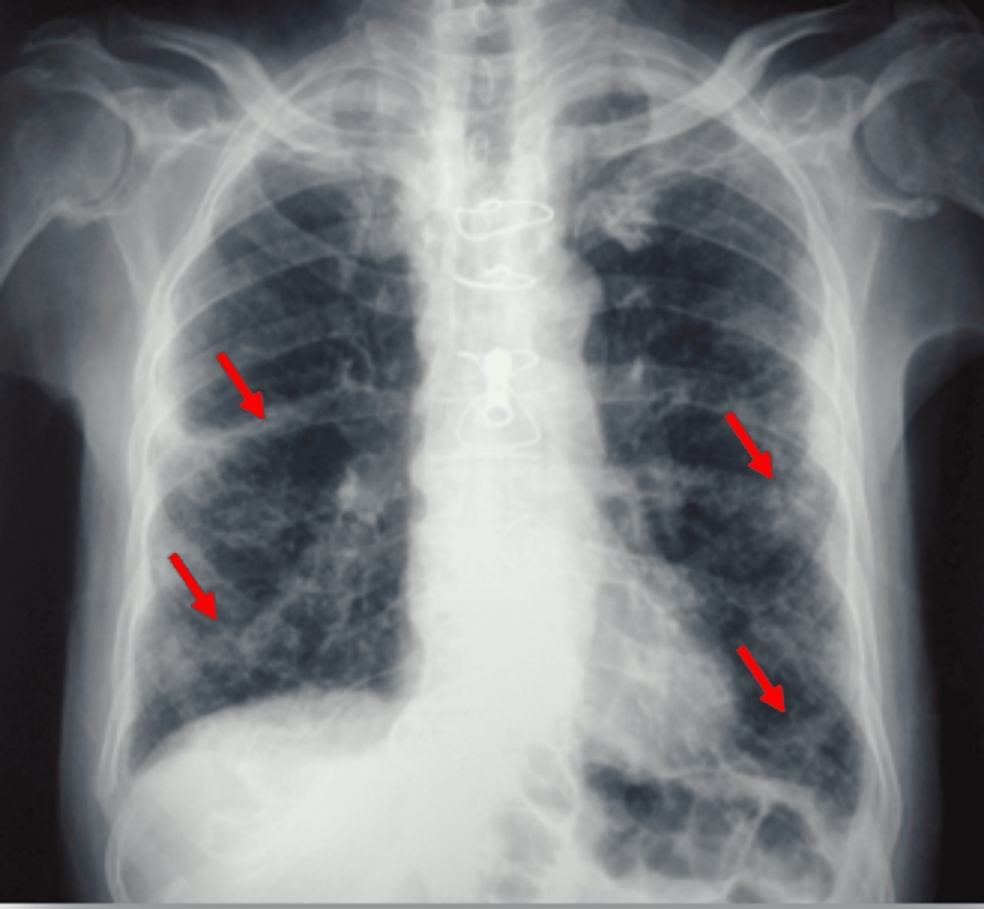
Thorax radiography: interstitial lung disease Presence of reticulo-micronodular interstitial infiltrates (red arrows)

**Figure 2:**
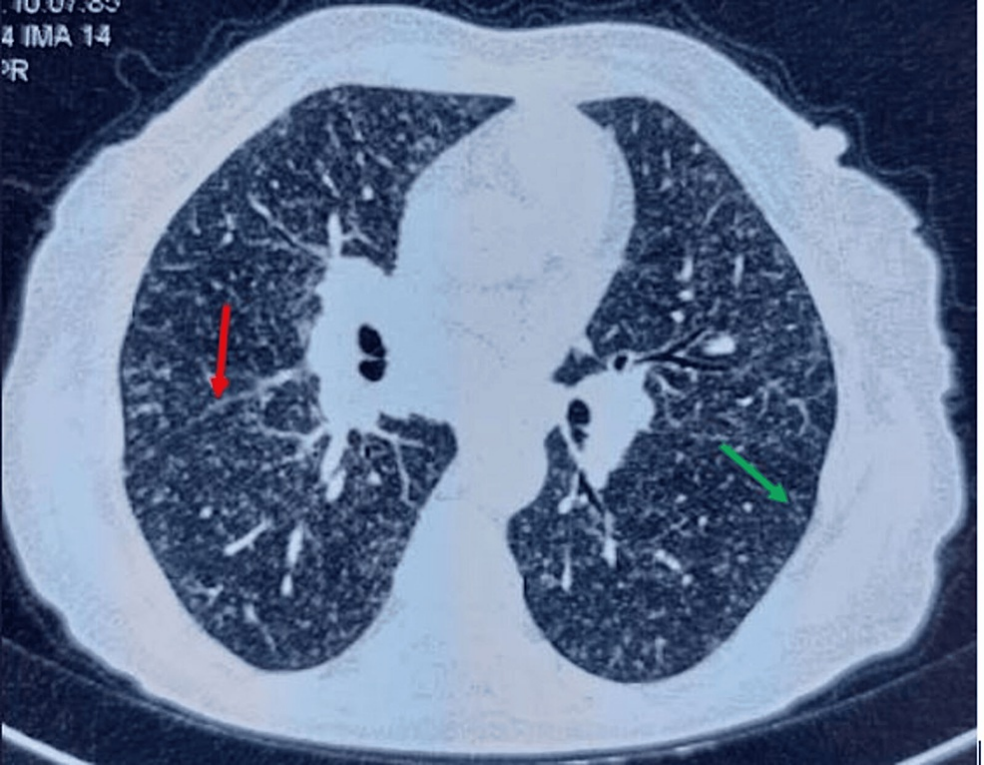
Thoracic CT scan (axial sections, parenchymal window) Micronodules with perilymphatic distribution at scissural (red arrow) and subpleural (green arrow) levels

**Figure 3:**
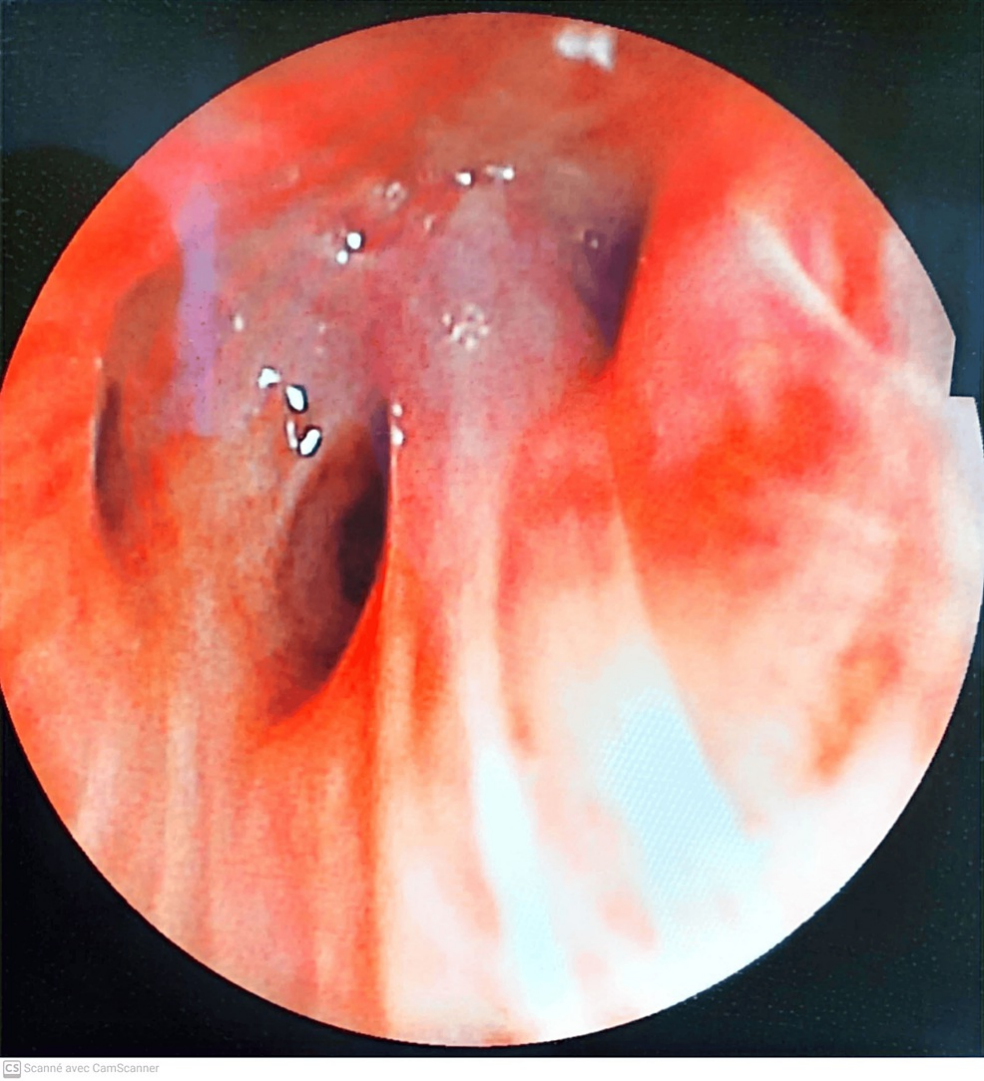
Thickening of bronchial spurs

**Figure 4:**
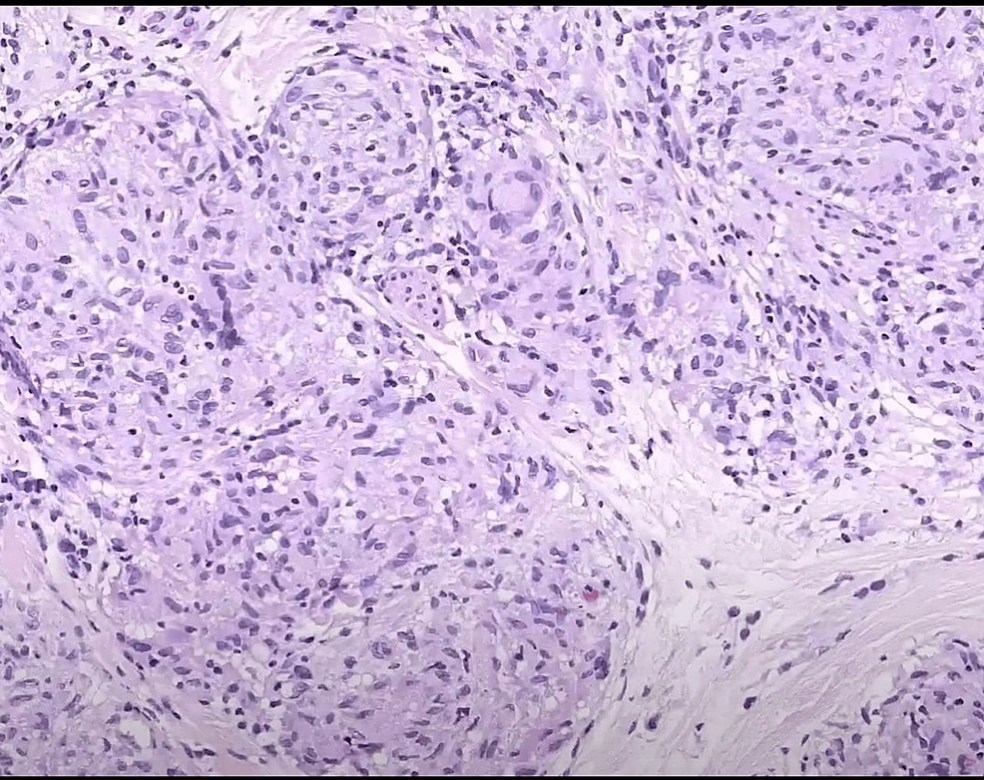
Histological examination showed a granulomatous reaction composed of multiple multinucleated giant cells and epithelioid cells admixed with some lymphocytes (H&E, x200).

In terms of the etiological investigation, the cerebral CT was normal, and the serum angiotensin-converting enzyme (ACE) level was high at 122 IU (Table 1). Serum protein electrophoresis (SPEP) showed hypergammaglobulinemia. Quantiferon was negative. A frontal chest X-ray showed bilateral interstitial syndrome (Figure 1). A CT scan of his chest (Figure 2) revealed micronodules with perilymphatic distribution at the scissural and subpleural levels and mediastinal adenomegalies. Bronchoscopy showed spur thickening (Figure 3), and spur biopsy was consistent with granuloma compatible with sarcoidosis (Figure 4). He was diagnosed with pulmonary sarcoidosis based on the presence of a granuloma on the bronchial biopsy and the absence of other causes of granuloma. Steroids were started (40 mg/day), with potassium supplementation and gastric protection, which improved his hypercalcemia.

As part of the impact assessment, the pulmonary plethysmography showed no obstructive or restrictive ventilatory disorder. Gasometry was normal, as was the fundoscopic examination.

## Discussion

Sarcoidosis is a systemic disease with unknown etiology, histologically characterized by noncaseating granulomas. It is seen all over the world, with an estimated prevalence of 20 to 60 cases per 100,000 people [6]. Hypercalcemia incidence in sarcoidosis is assessed differently; it varies between 2% and 63% based on the literature reviews [7, 8]. McCort et al. reported the highest incidence at 63% [9]. In their review of 509 patients, Mayock et al [10] observed a frequency of 17%. Taylor et al [11] similarly reported comparable results in their study involving 345 sarcoidosis patients. The frequency also varies according to sex and race [12].

Diagnosis becomes challenging when hypercalcemia is the main symptom because it may mimic various granulomatous diseases and malignancies. Diagnosis is based on a clinically and radiologically compatible presentation with at least one noncaseating granuloma. Elevated angiotensin-converting enzyme (ACE) levels are seen in 75% of patients with sarcoidosis [13].

The pathophysiological mechanism of this hypercalcemia is based on the existence of hypervitaminosis D. There is an inappropriate production of 1,25 dihydroxy-vitamin D by the granuloma due to an increase in the activity of one-alpha-hydroxylase, located in the monocluded cells of the granuloma, the enzyme enabling the conversion of 25-hydroxy-vitamin D into its active form, 1,25 dihydroxy-vitamin D. This leads to intestinal calcium hyperabsorption, hypercalciuria, and hypercalcemia. It should be noted that one-alpha-hydroxylase activity in macrophages differs from that expressed in renal tubule cells, as it is not regulated by calcitriol and parathyroid hormone (PTH) concentrations. It is inhibited by glucocorticoids [14].

Treatment includes lifestyle and dietary measures, such as the reduction of dietary calcium intake, the eviction of occult intakes of vitamin D or calcium-modifying drugs, and the avoidance of sun exposure. The suggested effect of bisphosphonates is the reduction of plasma calcium without reduction of 1,25-(OH)2-D or ACE, showing that hypercalcemia is secondary to the effect of 1,25-(OH)2-D on bone and that bisphosphonates do not directly affect granuloma activity [15]. The use of diuretics is not recommended in the absence of malignant hypercalcemia (>3.7 mmol/L) [16]. Treatment remains essentially medicinal and consists mainly of corticosteroids, which play an important role in managing hypercalcemia due to their direct effect on calcium and phosphate metabolism by reducing the absorption of calcium from the gastrointestinal tract, inhibiting osteoclast activity and inhibiting macrophage one-hydroxylase activity as an immunosuppressive effect. They may also transform 1,25-dihydroxyvitamin D3 into an inactive metabolite [17]. Intravenous hydration is proposed as an additional supportive treatment. Ketoconazole may be effective in treating hypercalcemia by inhibiting cytochrome P450 enzymes, including one-hydroxylase, in cases of steroid-resistant sarcoidosis [18].

## Conclusions

This case report shows a particular presentation of sarcoidosis, as malignant hypercalcemia is rarely the initial manifestation. The use of bisphosphonates, in association with the classic treatment of hypercalcemia by hyperdiuresis and corticosteroid, is a therapeutic option in cases of severe hypercalcemia complicating sarcoidosis.
